# An Ig Nobel for Global Health: The case for laughing at ourselves

**DOI:** 10.1371/journal.pgph.0007569

**Published:** 2026-10-29

**Authors:** Dhananjaya Sharma

**Affiliations:** Department of Surgery, NSCB Government Medical College, Jabalpur, Madhya Pradesh, India; PLOS: Public Library of Science, UNITED STATES OF AMERICA

The *Ig Nobel Prize*, a spoof on Nobel Prize, has long performed a valuable service to science. It honours achievements that make people laugh and then think [1]. Global Health, after several decades of earnest endeavour, would seem ready for similar recognition. I therefore propose, with all due humility, the establishment of an *Ig Nobel Prize for Global Health*.

There is, of course, no intention to mock Global Health, which is too noble, and the inequalities it seeks to address too consequential. But, like most human enterprises, Global Health has acquired its own vocabulary, rituals, paradoxes and unintended ironies. The proposed Ig Nobel Prize for Global Health would therefore recognise such phenomena for their ability to make us laugh first, think second, and perhaps recognise a little of ourselves in the process.

Five examples readily come to mind.

1. Designing ever more elaborate Global Health reforms

There is a certain elegance to the way Global Health reforms respond to complexity. Complex problems call for architecture. Architecture calls for frameworks. Frameworks call for coordination. Coordination calls for a task force. Task forces call for oversight. Oversight, naturally, calls for panels. And panels, sooner or later, require strategic direction. The result is an impressive ecosystem of structures: a Presidential Council, a Circle of Guardians, a High-Level Panel on Reform of the Global Health Architecture and Governance, and a 25-member Joint Task Force [2]. Each has an appropriately serious name, a worthy purpose and, presumably, a meeting schedule. It looks more like adding rooms to a building than reforming Global Health.

The ambition is admirable, the terminology impressive and the architecture ever-expanding. The proposed award should therefore recognise: “For demonstrating exceptional ingenuity in constructing an increasingly sophisticated Global Health reform architecture, demonstrating that when one structure is insufficient, another can always be convened.”

2. **The decolonisation–recolonisation paradox**

Global Health has rightly become increasingly concerned about colonial legacies and unequal power. We now have an impressive vocabulary for discussing them: decolonisation, localisation, power asymmetry, epistemic justice, equitable partnership and North–South imbalance. This is undoubtedly progress [3]. But there is an awkward sequel.

What happens when the formerly colonised acquire enough institutional power to reproduce some of the behaviours they once criticised? Could colonialism, like a microorganism with a well-developed survival instinct, simply adapt to its new environment and simply change hosts? [4] The symptoms can be remarkably familiar. The language becomes more inclusive; the geography more representative; but decolonisation does not always eliminate hierarchy. Sometimes it merely updates its address and those who were once excluded acquire a seat at the head of table and these neo-colonizers start deciding who sits at the table and who controls the agenda and minutes.

The proposed award should therefore recognise: *“For demonstrating exceptional adaptability in the survival of colonial behaviour, successfully overcoming decolonisation by changing its host, vocabulary and the postal address.”*

3. Riding the equity bandwagon without leaving the parking lot

Equity has become one of the most popular destinations in Global Health. It appears in titles, abstracts, conference themes, strategic plans, and mission statements. The vocabulary has become remarkably sophisticated [5]. It requires not only discussion but also changes in power, authorship, funding, decision-making, access and opportunity [6]. Interestingly, it is entirely possible to become an accomplished traveller on the equity bandwagon without actually leaving the parking lot [7]. Hence the emergence of a distinct academic discipline: *Equity Theatre***.** Put “equity” on the agenda. Organise a panel with a representative from the Global South to speak about inequity. Publish the obligatory photograph and proceedings. Develop a framework explaining why progress remains difficult. The same framework can be used to measure the equity deficit, demonstrate commitment to addressing it, and justify another framework. Everyone agrees that considerable progress has been made, and thus we arrive at a remarkable academic innovation: *equity without redistribution*.

The proposed award should therefore recognise: “*For outstanding achievement in advancing equity discourse over considerable distances while demonstrating exceptional ability to remain exactly where one started*.”

4. Achieving Global Health impact in seven days

Global Health has also demonstrated an admirable confidence in the transformative potential of short interventions to produce long-term transformation. The modern short-term mission trip may last seven days, while its sustainability plan may extend for ten years. The photograph documenting the intervention may last forever. The itinerary is reassuringly simple: Day 1: arrive. Day 2: assess. Day 3: teach. Day 4: operate. Day 5: build capacity. Day 6: discuss sustainability. Day 7: depart. The subsequent ten-year follow-up is, naturally, subject to funding [8].

There is nothing inherently wrong with short-term missions [9]. Many partnerships can provide genuine benefit [10]. The satire begins when a brief visit acquires a remarkably long institutional afterlife. Seven days can apparently lead to an extraordinary scientific achievement: generate capacity building, sustainable partnership, health-system strengthening, knowledge transfer, cultural exchange and transformative impact. The mission may be short, but its adjectives are not. This creates a particularly elegant form of Global Health mathematics: the shorter the visit, the longer the sustainability plan. The final day may therefore be devoted to discussing how the work will continue for the next decade, preferably after the visiting team has returned home.

The proposed award should therefore recognise: “*For demonstrating exceptional ability to convert seven days of intervention into ten years of sustainability, preferably before boarding the return flight*.”

5. **The visiting professor phenomenon: After he/ she leaves**

The visiting professor phenomenon occupies a special place in Global Health. Professor arrives from afar, usually carrying a PowerPoint presentation, considerable goodwill and a lifetime of accumulated expertise. The hosts are delighted to welcome the visitor. A lecture is delivered. A group photograph, a certificate, a plaque and a ceremonial dinner follow. For a brief period, academic exchange acquires the atmosphere of a state occasion.

Then the professor leaves [11]. Photographs, certificates and plaques are easier to document. Sustainability, mentorship, capacity building and enduring collaboration are somewhat less tangible and the real measure is whether the academic footprint extends beyond the visit.

The proposed award should therefore recognise: *“For demonstrating that a visiting professorship can be successfully completed with great academic ceremony, memorable photographs and an impressive collection of certificates, while demonstrating exceptional confidence that some tangible benefit actually remains after the professor has gone home.”*

## Why Global Health might merit an Ig Nobel

It is not a surprise that Global Health has accumulated contradictions. Every serious human enterprise does. What is remarkable is that we have become remarkably accomplished at giving those contradictions elegant names, impressive architectures and carefully considered strategic plans.

The five examples above are, of course, exaggerated. That is what satire does. Yet exaggeration works best when it has something recognisable to exaggerate. The uncomfortable part is not that the examples are entirely true. It is that they are not entirely untrue. The real value of satire does not lie in laughing at Global Health, but in giving those of us who inhabit it permission to laugh at ourselves. The prize, therefore, can remain imaginary. The invitation to look in the mirror need not be.
